# Clinical characteristics and trends in the antimicrobial susceptibility profile of *Streptococcus suis* infections in a large tertiary hospital, Thailand, 2007–2023

**DOI:** 10.1371/journal.pntd.0013110

**Published:** 2025-05-19

**Authors:** Chittamas Panpaeng, Witchuda Kamolvit, Khemajira Karaketklang, Anupop Jitmuang

**Affiliations:** 1 Department of Medicine, Faculty of Medicine Siriraj Hospital, Mahidol University, Bangkok, Thailand; 2 Department of Microbiology, Faculty of Medicine Siriraj Hospital, Mahidol University, Bangkok, Thailand; Yale University School of Medicine, UNITED STATES OF AMERICA

## Abstract

**Background:**

*Streptococcus suis* is an emerging pathogen causing invasive zoonotic infections in humans. Antimicrobial resistance (AMR) to penicillin, macrolides, and tetracyclines has emerged in *S. suis*-infected swine. AMR among zoonotic *S. suis* strains causes a critical concern for human infection and antimicrobial treatment options. Thus, the study aims to delineate the clinical characteristics and to explore the changing pattern of the antimicrobial susceptibility (AST) of *S. suis* infection in humans.

**Methods:**

We conducted a chart review of adult patients with culture-confirmed *S. suis* infection in any body sites at Siriraj Hospital, Bangkok, Thailand, between May 2007 and May 2023. We also reviewed the AST profile of *S. suis* isolates during the study period.

**Results:**

Over 16 years, 86 adult patients with *S. suis* infection were identified (59.3% male, mean age 59.29 ± 14.46 years). Of them, 60.5% had comorbidities (hypertension 43%, dyslipidemia 23.3%, diabetes mellitus 20.9%, alcoholism 19.8%), and 35.0% had swine contact a median of 1 (0.0-6.5) days before onset. Clinical presentations included septicemia (91.9%), meningitis (30.2%), endocarditis (26.7%), and septic arthritis (9.3%), leading to multiorgan dysfunction (32.5%), cardiopulmonary failure (26.8%), renal failure (17.4%), and septic shock (14.0%). Mortality was 7%. Definitive therapy primarily used ceftriaxone (76.7%) or penicillin (8.1%) as a basis regimen. Among *S. suis* isolates tested, 48.2% were susceptible to penicillin (median MIC 0.064 [0.047–0.094] µg/mL), 96.5% were susceptible to ceftriaxone (median MIC 0.380 [0.110–0.500] µg/mL), and susceptibility to vancomycin, ofloxacin, tetracyclines, and clindamycin was 100%, 96.4%, 4.8%, and 1.2%, respectively. Penicillin MICs increased significantly (p < 0.001), while other agents’ profiles remained stable.

**Conclusions:**

*S. suis* can cause severe human infection, leading to fatal complications. *S. suis* displayed an upward trend of penicillin MICs and resistance to several antimicrobial agents. These findings underscore the need to monitor emerging resistance.

## Author summary

*Streptococcus suis* is an emerging pathogen causing zoonotic infection in humans. Antimicrobial resistance (AMR) to antibiotics such as penicillin, macrolides, and tetracyclines has emerged in *S. suis*-infected swine. *S. suis* human infection can cause severe disease among healthy populations, and conventional penicillin has been recommended as the first-line treatment for several decades. However, AMR among zoonotic *S. suis* strains causes a critical concern for human infection and antimicrobial treatment options. Our study demonstrated *S. suis* commonly causes bacteremia, meningitis, and endocarditis in humans, and only one-third of cases had apparent exposure to swine before the onset. Multiorgan dysfunction (MOD) is the leading cause of fatal outcomes. In contrast to previous studies, *S. suis* exhibited 48.2% penicillin susceptibility with an increasing trend of penicillin MICs. Our findings underline a reconsideration of the appropriate antimicrobial agent to treat this infection.

## Introduction

*Streptococcus suis* infection is an emerging zoonotic infectious disease caused by gram-positive bacteria that commonly colonize the upper airways of swine, particularly the tonsils and nasal cavities [1]. It usually causes severe and fatal infections, mainly in piglets, and can be transmitted to humans through contact with infected pigs [2,3]. *S. suis* human infection gained international attention when a large outbreak occurred in China in 2005, where 215 cases were reported, resulting in 39 fatalities with a mortality rate of 18% [4]. Several studies have identified common risk factors for *S. suis* infection in humans, including being male, having occupational contact with pigs, or consuming undercooked pork and pig-derived products [5,6]. As pig farming and pork consumption are customary in China and Southeast Asia, especially in Thailand and Vietnam, the incidence of this infection is relatively high in these regions [5].

In Thailand, *S. suis* human infection cases have been reported since 1987. The Bureau of Epidemiology, Ministry of Public Health, Thailand, reported 468 cases of *S*. *suis* infection in humans in 2021, resulting in an incidence rate of 0.70 per 100 000 people and a mortality rate of 0.03 per 100 000 people in 2021 [7]. *S. suis* can cause severe diseases, such as acute meningitis, bacteremia, and endocarditis, in healthy populations. Severe infection can cause multiorgan dysfunction, septic shock, and disseminated intravascular coagulation, leading to fatal outcomes [5,8–12]. The mortality rate of this particular infection has ranged between 10% and 40% in various studies [5,8–11]. Additionally, the *S. suis* serotypes and delayed bacterial eradication pose potential risks for mortality [1,8].

Strains of *S. suis* isolated from humans in Thailand have been previously reported to exhibit high susceptibility to penicillin (90–100%) and 100% susceptibility to ceftriaxone and cefepime [11,13]. This indicates that penicillin or cephalosporins have been previously employed as the first-line treatment for *S. suis* infection [14]. Furthermore, all the isolates were susceptible to vancomycin, and almost all (99.6%) were susceptible to levofloxacin [8,13,15]. Antimicrobial resistance is a growing concern for this infection. Pig-isolated strains are resistant to several antibiotics, such as penicillin (27.0%), ampicillin (21.7%), cefotaxime (4.2%), clindamycin (96.3%), erythromycin (83.0%), ciprofloxacin (31.7%), and levofloxacin (26.5%) [15]. These findings suggest that humans may also become infected with these strains, which can lead to limited treatment options due to the development of antimicrobial resistance. It is essential to be vigilant and take necessary precautions to prevent the transmission of antimicrobial-resistant strains that cause human infection.

Thus, this study aimed to investigate the clinical characteristics and treatment outcomes of *S. suis* infection and to monitor the evolving trends in the antimicrobial susceptibility of *S. suis* infection in humans. This information can aid in establishing guidelines for managing and treating *S. suis* infections in the future.

## Results

We identified 86 cases of *S. suis* infection during the study period, 59.3% male. The mean age was 59.29 ± 14.46 years. Most cases (79.1%) involved domiciles in the central region of Thailand. Table 1 shows the frequent underlying medical conditions among patients with known comorbidities (60.5%). Among 80 patients, 35.0% had potential risks or exposures, such as consuming undercooked pork or pig-derived products (16.3%), handling raw meat (8.8%), and occupational exposure (10%). Moreover, 65% had unknown risk factors. The patients experienced symptoms quickly after exposure to the bacteria, with the average time for symptom onset being 1 (0–6.5) day. In this study, septicemia was the most common manifestation of *S. suis* infection (91.9%), followed by acute meningitis (30.2%), endocarditis (26.7%), septic arthritis (9.3%), and spondylodiscitis (9.3%), as shown in Table 1. Of the 23 cases of infective endocarditis, the echocardiographic findings indicated that endocarditis affected the aortic valves in 12 patients, the mitral valves in 9 patients, and both aortic and mitral valves in 2 patients. 15 of those patients required heart valve reconstruction and replacement to fix severe cardiac dysfunction. *S. suis* infection can lead to severe complications from individual organ dysfunction, such as cardiopulmonary failure (26.8%) and renal failure (17.4%) to the presence of multiorgan dysfunction (32.5%), which includes septic shock, acute renal failure, cardiopulmonary failure, and disseminated intravascular coagulation. The study found five patients developed significant neurological deficits due to major CNS complications after the onset (Table 1). The major CNS complications, diagnosed using CT scans and MRI studies, included spinal epidural abscesses in three cases with *S. suis* spondylodiscitis, a large cerebral infarction with hemorrhagic transformation in one case with fatal *S. suis* endocarditis, and seizures in one case of acute meningitis. Among the 34 patients who underwent CSF examination, 26 were diagnosed with acute meningitis based on the study criteria. Of these, 17 had *S. suis* isolated from their CSF cultures, whereas only 7 had a positive Gram stain from the CSF sample. The other CSF profiles and additional laboratory findings are presented in Table 1. *S. suis* infection was usually diagnosed by positive blood cultures, which accounted for 91.8% of cases. Tissue culture was also used for diagnosis but accounted for only 34.1% of the cases.

**Table 1:** Baseline characteristics, clinical data, and treatment outcomes of *S. suis* infection.

Variable	Total (n = 86)
**Gender,** n (%)	
Male	51 (59.3)
Female	35 (40.7)
Age (years), mean ± SD	59.29 ± 14.46
BMI (kg/m^2^), mean ± SD	23.47 ± 3.99
**Places of domicile in Thailand,** n (%)	
Central region	68 (79.1)
Northeastern region	9 (10.5)
Northern region	4 (4.7)
Eastern region	4 (4.7)
Southern region	1 (1.2)
**Underlying disease,** n (%)	
Hypertension	37 (43.0)
Dyslipidemia	20 (23.3)
Diabetes mellitus	18 (20.9)
Alcoholism	17 (19.8)
Cardiovascular disease	10 (11.6)
Cerebrovascular disease	7 (8.1)
HIV infection	6 (7.0)
Liver disease	5 (5.8)
Malignancy	5 (5.8)
Chronic kidney disease	4 (4.7)
Splenectomy	2 (2.3)
Steroid used	2 (2.3)
None	34 (39.5)
**Potential risk or exposure,** n (%)	n = 80
Ingesting undercooked pork or pig-derived products	13 (16.3)
Preparing or handling raw meat	7 (8.8)
Occupational exposures (butcher, slaughterer, pig farmer)	8 (10.0)
Unknown	52 (65.0)
Time from exposure to symptom onset (days), median (IQR)	1 (0.0-6.5)
**Patient care unit per clinical status at the onset,** n (%)	
General ward	66 (76.7)
Triage or out-patient unit	11 (12.8)
Intensive care unit	9 (10.5)
**Clinical manifestations,** n (%)	
Fever	77 (89.5)
Respiratory distress^a^	14 (16.3)
Alteration of consciousness	13 (15.1)
Headache	10 (11.6)
Neck stiffness	8 (9.3)
Diarrhea	8 (9.3)
Back pain	8 (9.3)
Neurological deficits (seizure, motor weakness)	7 (8.1)
Abdominal pain	5 (5.8)
Vomiting	4 (4.7)
Joint pain	4 (4.7)
Blurred vision	2 (2.3)
SOFA score at the onset, median (IQR)	1 (0.0-4.0)
**Clinical infections,** n (%)	
Septicemia	79 (91.9)
Acute meningitis	26 (30.2)
Endocarditis	23 (26.7)
Septic arthritis	8 (9.3)
Spondylodiscitis	8 (9.3)
Endophthalmitis	2 (2.3)
Pneumonia	1 (1.2)
Spontaneous bacterial peritonitis	1 (1.2)
**Complications,** n (%)	
Multiorgan dysfunction^b^	28 (32.5)
Cardiopulmonary failure	23 (26.8)
Renal Failure	15 (17.4)
Septic shock	12 (14.0)
Disseminated intravascular coagulation	6 (7.0)
Subsequent co-infections^c^	6 (7.0)
Major CNS complications^d^	5 (5.8)
None	37 (43.0)
**Laboratory findings at the onset**	
Hemoglobin (g/dL), mean ± SD	11.90 ± 2.37
White blood cell count (cells/µL), median (IQR)	13840 (9740-18055)
Neutrophil (%), mean ± SD	83 ± 10.94
Lymphocyte (%), median (IQR)	9 (5.3-13.7)
Platelet count (x10^3^ cells/µL), median (IQR)	188 (127.0-236.0)
BUN (mg/dL), median (IQR)	14.50 (11.1-22.1)
Creatinine (mg/dL), median (IQR)	0.87 (0.71-1.30)
Total bilirubin (mg/dL), median (IQR)	0.7 (0.5-1.1)
Direct bilirubin (mg/dL), median (IQR)	0.34 (0.20-0.67)
Albumin (g/dL), mean ± SD	3.32 ± 0.58
Globulin (g/dL), mean ± SD	3.87 ± 0.84
AST (U/L), median (IQR)	39 (23.0-75.0)
ALT (U/L), median (IQR)	33 (19.0-67.5)
ALP (U/L), median (IQR)	102.50 (72.25-133.50)
PT (second), median (IQR) (n = 39)	13.20 (11.90-15.30)
PTT (second), median (IQR) (n = 38)	27.90 (25.82-30.77)
INR, median (IQR) (n = 18)	1.12 (1.02-1.28)
Fibrinogen (mg/dL), median (IQR) (n = 10)	345.75 (109.77-486.80)
D-Dimer (ng/mL), median (IQR) (n = 6)	9033.47 (2947-16300)
CRP (mg/dL), median (IQR) (n = 13)	110.85 (45.5-136.51)
**CSF findings**	n = 34
White cell count (cells/µL), median (IQR)	480 (13.25-1538.75)
Neutrophil (%), median (IQR)	70 (3-87.5)
Lymphocyte (%), median (IQR)	13 (3.75-40.5)
CSF and blood glucose ratio (mg/dL), median (IQR)	0.34 (0.12-0.45)
Protein (mg/dL), median (IQR)	202 (51.75-350.75)
Gram stain positive, n (%)	7 (20.6)
CSF culture positive, n (%)	17 (50.0)
**Microbiological diagnoses,** n (%)	
Blood culture positive (n = 85)^e^	78 (91.8)
Tissue culture positive (n = 41)	14 (34.1)
Molecular identification assay (n = 3)	3 (100.0)
**Primary antimicrobial therapy,** n (%)	
Ceftriaxone	59 (68.6)
Carbapenems	7 (8.1)
Ceftriaxone combined with ampicillin	5 (5.8)
Penicillin G combined with gentamicin	4 (4.7)
Penicillin G	3 (3.5)
Vancomycin	3 (3.5)
Ceftriaxone combined with gentamicin	2 (2.3)
Others	3 (3.5)
Treatment duration (days), median (IQR)	21 (7.0-28.0)
**Maintenance oral antimicrobial therapy,** n (%)	n = 30
Fluoroquinolones	19 (63.3)
Amoxicillin-clavulanate	5 (16.7)
Amoxicillin	4 (13.3)
Others	2 (2.4)
Treatment duration (days), median (IQR)	10 (7.0-42.0)
Total course of antimicrobial therapy (days), median (IQR)	21 (14.0-28.0)
Surgical managements, n (%)	23 (26.7)
Adjunctive dexamethasone therapy, n (%)	7 (8.1)
Duration of hospital stay (days), median (IQR)	13.50 (7.0-25.0)
**Outcomes,** n (%)	
Complete recovery	61 (70.9)
Partial recovery with sequelae	19 (22.1)
Hearing impairment^f^	13 (15.1)
Vestibular dysfunction	2 (2.3)
Coexisting vestibular dysfunction and hearing impairment	2 (2.3)
Visual loss	1 (1.2)
Paraplegia	1 (1.2)
Death	6 (7.0)

Abbreviations: ALP, alkaline phosphatase; ALT, alanine transaminase; AST, aspartate transaminase; BMI, body mass index; BUN, blood urea nitrogen; CNS, central nervous system; CRP, C-reactive protein; CSF, cerebrospinal fluid; HIV, human immunodeficiency virus; INR, international normalized ratio; IQR, interquartile range; PT, prothrombin time; PTT, partial thromboplastin time; SOFA, sequential organ failure assessment.

^a^Respiratory distress is defined as the development of signs such as shortness of breath or increased respiratory effort at the onset, including a respiratory rate >22 breaths per minute, rapid and deep breathing, the presence of chest wall retraction, or oxygen desaturation that necessitates oxygen therapy.

^b^Multiorgan dysfunction (MOD) is defined as the identification of at least two organ dysfunctions, such as septic shock, acute renal failure, cardiopulmonary failure, and disseminated intravascular coagulation.

^c^Subsequent co-infections included hospital-acquired pneumonia, ventilator-associated pneumonia, nosocomial diarrhea, catheter-related urinary tract infection, and catheter-related bloodstream infection.

^d^The following are considered major complications of the central nervous system (CNS): spinal epidural abscess, cerebral infarction and hemorrhagic transformation, and seizure.

^e^Based on the physician’s discretion at the time, one patient with a localized infection did not undergo blood culture sampling.

^f^Including patients with any hearing impairment (permanent or temporary, in one or both ears).

Among the 86 patients infected with *S. suis*, 59 (68.6%) received intravenous ceftriaxone as the primary antimicrobial therapy for an average of 21 days. Thirty patients required additional oral antimicrobial agents, such as fluoroquinolones (63.3%), amoxicillin-clavulanate (16.7%), and amoxicillin (13.3%), as maintenance therapy for an average of 10 days. The total duration of antimicrobial therapy ultimately averaged 21 (14–28) days. Twenty-three patients with endocarditis, septic arthritis, spondylodiscitis, and endophthalmitis underwent surgical management to eradicate the infectious foci. Seven (8.1%) patients received adjunctive dexamethasone for acute meningitis, but four of them experienced hearing impairment. After infection with *S. suis*, 61 out of 86 patients (70.9%) fully recovered. However, 19 patients (22.1%) who achieved partial recovery still experienced neurological sequelae, such as hearing impairment (15.1%) and vestibular dysfunction (2.3%). Unfortunately, six patients (7%) had fatal outcomes due to septicemia with severe complications, as shown in Tables 1 and S1.

Among the six patients who had fatal outcomes due to *S. suis* septicemia, five were male, and their ages ranged from 33 to 72 years, as shown in S1 Table. Two of these patients were healthy and had no known underlying disease. All patients had elevated sequential organ failure assessment (SOFA) scores of 2 or more at onset, corresponding to an increased risk of sepsis mortality. Most deadly *S. suis* strains were susceptible to penicillin and ceftriaxone, except for one strain that showed intermediate susceptibility to both antibiotics. Five patients died rapidly due to severe complications, while one patient had a prolonged intensive care unit stay and subsequent nosocomial infections before dying.

### Factors associated with multiorgan dysfunction in *S. suis* infection

Patients diagnosed with MOD were found to have significantly greater rates of younger age, respiratory distress and gastrointestinal symptoms, elevated SOFA scores, endocarditis, and subsequent co-infections. On the other hand, patients with *S. suis* meningitis had a significantly lower rate of MOD (Table 2). Independent factors strongly associated with MOD in *S. suis* infection patients were respiratory distress (odds ratio [OR] 57.694, 95% confidence interval [CI] 3.129–1063.805; *p* = 0.006), an elevated SOFA score (OR 3.424, 95% CI 1.805–6.495; *p* < 0.001), and subsequent co-infection (OR 89.167, 95% CI 2.247–3538.090; *p* = 0.017; Table 3).

**Table 2:** Comparison of baseline characteristics, clinical data, management, and treatment outcomes of *S. suis* infection between patients with and without multiorgan dysfunctions (MOD).

Variable	MOD group^a^ (n = 28)	Non-MOD group (N = 58)	*p*-value
**Gender,** n (%)			0.853
Male	17 (60.7)	34 (58.6)	
Female	11 (39.3)	24 (41.4)	
Age (years), mean ± SD	53.89 ± 13.64	61.90 ± 14.24	0.015
BMI (kg/m2), mean ± SD	23.39 ± 3.41	23.52 ± 4.27	0.896
**Underlying disease,** n (%)			
Hypertension	10 (35.7)	27 (46.6)	0.342
Alcoholism	8 (28.6)	9 (15.5)	0.154
Dyslipidemia	5 (17.9)	15 (25.9)	0.410
Diabetes mellitus	4 (14.3)	14 (24.1)	0.293
HIV infection	3 (10.7)	3 (5.2)	0.386
Cardiovascular disease	2 (7.1)	8 (13.8)	0.488
Cerebrovascular disease	2 (7.1)	5 (8.6)	1.000
Liver disease	2 (7.1)	3 (5.2)	0.659
Splenectomy	2 (7.1)	0 (0.0)	0.103
Malignancy	1 (3.6)	4 (6.9)	1.000
Chronic kidney disease	1 (3.6)	3 (5.2)	1.000
Steroid Used	1 (3.6)	1 (1.7)	0.548
No	13 (46.4)	21 (36.2)	0.364
**Clinical manifestations,** n (%)			
Fever	26 (92.9)	51 (87.9)	0.712
Respiratory distress^b^	12 (42.9)	2 (3.4)	<0.001
Diarrhea	6 (21.4)	2 (3.4)	0.013
Abdominal pain	4 (14.3)	1 (1.7)	0.037
Alteration of consciousness	4 (14.3)	9 (15.5)	1.000
Neurological deficits (seizure, motor weakness)	2 (7.1)	5 (8.6)	1.000
Joint pain	2 (3.4)	2 (3.4)	0.593
Vomiting	2 (7.1)	2 (3.4)	0.593
Back pain	1 (3.6)	7 (12.1)	0.265
Neck stiffness	1 (3.6)	7 (12.1)	0.265
Headache	0 (0.0)	10 (17.2)	0.027
Blurred vision	0 (0.0)	2 (3.4)	1.000
SOFA score at the onset, median (IQR)	4 (2.5-6.0)	0 (0.0-2.0)	<0.001
**Clinical infections,** n (%)			
Septicemia	26 (92.9)	53 (91.4)	1.000
Endocarditis	12 (42.9)	11 (19.0)	0.019
Septic arthritis	4 (14.3)	4 (6.9)	0.429
Acute meningitis	3 (10.7)	23 (39.7)	0.006
Pneumonia	1 (3.6)	0 (0.0)	0.326
Spondylodiscitis	0 (0.0)	8 (13.8)	0.049
Endophthalmitis	0 (0.0)	2 (3.4)	1.000
Spontaneous bacterial peritonitis	0 (0.0)	1 (1.7)	1.000
**Neurological complications,** n (%)			
Hearing impairment^c^	3 (10.7)	12 (20.7)	0.366
Major CNS complications^d^	1 (3.6)	4 (6.9)	1.000
Vestibular dysfunction^c^	0 (0.0)	4 (6.9)	0.299
Visual loss	0 (0.0)	1 (1.7)	1.000
Subsequent co-infections^e^	5 (17.9)	1 (1.7)	0.013
**Laboratory findings**			
Hemoglobin (g/dL), mean ± SD	12.15 ± 2.30	11.78 ± 2.42	0.507
White blood cell count (cells/µL), median (IQR)	15240 (10260-19160)	13635 (8960-16630)	0.423
Neutrophil (%), mean ± SD	82.09 ± 12.16	83.59 ± 10.39	0.556
Lymphocyte (%), median (IQR)	10.55 (3.75-15.65)	8.5 (5.70-13.10)	0.558
Platelet count (x 10^3^ cells/µL), median (IQR)	178 (101.7-237)	199.5 (145-268)	0.167
BUN (mg/dL), median (IQR)	20.50 (11.65-27.10)	14.00 (11.20-20.40)	0.082
Creatinine (mg/dL), median (IQR)	1.08 (0.74-1.72)	0.85 (0.72-1.06)	0.105
Total bilirubin (mg/dL), median (IQR)	0.84 (0.65-1.36)	0.70 (0.41-1.00)	0.160
Direct bilirubin (mg/dL), median (IQR)	0.38 (0.21-0.83)	0.34 (0.21-0.59)	0.614
Albumin (g/dL), mean ± SD	3.35 ± 0.54	3.31 ± 0.62	0.789
Globulin (g/dL), mean ± SD	3.60 ± 0.86	4.02 ± 0.81	0.043
AST (U/L), median (IQR)	38.50 (23-82)	40 (23-75)	0.773
ALT (U/L), median (IQR)	29 (21-64)	40 (18-76)	0.800
ALP (U/L), median (IQR)	96.50 (67-125)	108 (73-135)	0.656
PT (second), median (IQR) (n = 39)	13.50 (12.20-16.30)	12.65 (11.70-14.10)	0.133
PTT (second), median (IQR) (n = 38)	28.55 (27.70-36.70)	26.40 (24.40-28.70)	0.012
INR, median (IQR) (n = 18)	1.43 ± 0.65	1.02 ± 0.41	0.128
Fibrinogen (mg/dL), median (IQR) (n = 10)	191.40 (126.90-344.40)	373 (347.10-425.30)	0.465
D-Dimer (ng/mL), median (IQR) (n = 6)	9533 (6348-22600)	4950.47 (900.00-9000.94)	0.165
CRP (mg/dL), median (IQR) (n = 13)	118 (11.59-136.60)	85.25 (53.00-136.42)	1.000
**Microbiological diagnoses,** n (%)			
Blood cultures positive (n = 85)	26 (96.3)	52 (89.7)	0.423
CSF cultures positive (n = 34)	2 (50.0)	15 (50.0)	1.000
Tissue cultures positive (n = 41)	6 (35.3)	8 (33.3)	1.000
**Primary antimicrobial therapy,** n (%)			
Ceftriaxone	16 (57.1)	43 (74.1)	0.112
Penicillin	3 (10.7)	0 (0.0)	0.032
Ceftriaxone combined with ampicillin	3 (10.7)	2 (3.4)	0.324
Carbapenems	2 (7.1)	5 (8.6)	1.000
Vancomycin	1 (3.6)	2 (3.4)	1.000
Ceftriaxone combined with gentamicin	1 (3.6)	1 (1.7)	1.000
Penicillin G combined with gentamicin	0 (0.0)	4 (6.9)	0.299
Others	2 (7.1)	1 (1.7)	0.246
Treatment duration (days), median (IQR)	21 (7.0-28.0)	14 (10.0-28.0)	0.293
**Maintenance oral antimicrobial therapy,** n (%)			
Fluoroquinolones	4 (57.1)	15 (65.2)	1.000
Amoxicillin-clavulanate	2 (28.6)	3 (13.0)	0.565
Amoxicillin	1 (14.3)	3 (13.0)	1.000
Others	0 (0.0)	2 (8.7)	1.000
Treatment duration (days), median (IQR)	28 (7.0-180.0)	10 (7.0-40.0)	0.230
Total course of antimicrobial therapy (days), median (IQR)	28 (15.5-42.0)	21 (14.0-28.0)	0.153
**Antimicrobial susceptibility testing of the patient’s isolate,** n (%)			
Penicillin susceptible	17 (63.0)	26 (44.8)	0.296
Penicillin MICs (µg/mL), median (IQR)	0.11 (0.06-0.44)	0.16 (0.08-0.25)	0.454
Ceftriaxone susceptible	27 (100.0)	55 (94.8)	0.548
Ceftriaxone MICs (µg/mL), median (IQR)	0.38 (0.11-0.50)	0.38 (0.19-0.50)	0.381
**Outcomes, n (%)**			0.013
Overall recovery	23 (82.1)	57 (98.3)	
Death	5 (17.9)	1 (1.7)	

**Abbreviations:** ALP, alkaline phosphatase; ALT, alanine transaminase; AST, aspartate transaminase; BMI, body mass index; BUN, blood urea nitrogen; CNS, central nervous system; CRP, C-reactive protein; CSF, cerebrospinal fluid; HIV, human immunodeficiency virus; INR, international normalized ratio; IQR, interquartile range; MIC, minimum inhibitory concentrations; PT, prothrombin time; PTT, partial thromboplastin time; SOFA, sequential organ failure assessment.

^a^Multiorgan dysfunction (MOD) is defined as the identification of at least two organ dysfunctions, such as septic shock, acute renal failure, cardiopulmonary failure, and disseminated intravascular coagulation.

^b^Respiratory distress is defined as the development of signs such as shortness of breath or increased respiratory effort at the onset, including a respiratory rate >22 breaths per minute, rapid and deep breathing, the presence of chest wall retraction, or oxygen desaturation that necessitates oxygen therapy.

^c^Two patients had coexisting vestibular dysfunction and hearing impairment.

^d^The following are considered major complications of the central nervous system (CNS): spinal epidural abscess, cerebral infarction and hemorrhagic transformation, and seizure.

^e^Subsequent co-infections included hospital-acquired pneumonia, ventilator-associated pneumonia, nosocomial diarrhea, catheter-related urinary tract infection, and catheter-related bloodstream infection.

**Table 3:** Factors associated with multiorgan dysfunction in *S. suis* infection.

Variable	Univariate analysis		Multivariate analysis	
	OR (95%CI)	*p*-value	OR (95%CI)	*p*-value
Age	0.960 (0.928-0.993)	0.019		
Respiratory distress^a^	21.000 (4.254-103.674)	<0.001	57.694 (3.129-1063.805)	0.006
Diarrhea	7.636 (1.431-40.751)	0.017		
Abdominal pain	9.500 (1.009-89.469)	0.049		
SOFA score	2.178 (1.546-3.066)	<0.001	3.424 (1.805-6.495)	<0.001
Endocarditis	3.205 (1.184-8.672)	0.022		
Meningitis	0.183 (0.049-0.675)	0.011		
Subsequent co-infection^b^	12.391 (1.372-111.938)	0.025	89.167 (2.247-3538.090)	0.017
Globulin level	0.560 (0.303-1.036)	0.065		

Abbreviations: 95%CI, 95% confidence interval; OR, odds Ratio; SOFA, sequential organ failure assessment

^a^Respiratory distress is defined as the development of signs such as shortness of breath or increased respiratory effort at the onset, including a respiratory rate >22 breaths per minute, rapid and deep breathing, the presence of chest wall retraction, or oxygen desaturation that necessitates oxygen therapy.

^b^Subsequent co-infections included hospital-acquired pneumonia, ventilator-associated pneumonia, nosocomial diarrhea, catheter-related urinary tract infection, and catheter-related bloodstream infection.

### Antimicrobial susceptibility profile of *S. suis* isolates throughout the study period

**Fig 1:**
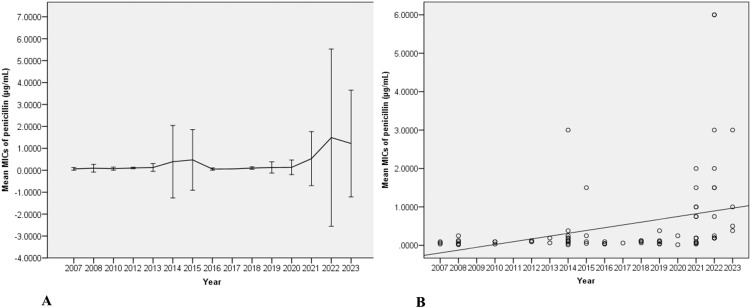
(A) A graphical representation indicating that the mean minimal inhibitory concentrations (MICs) of penicillin against *S. suis* isolates significantly increased over the last 4 years of the study period. (B) A significant trend in the mean MICs of penicillin against the isolates throughout the study period (p < 0.001).

Table 4 displays the results of antimicrobial susceptibility testing conducted on non-duplicated *S. suis* isolated from patients between 2007 and 2023. Among the isolates tested for penicillin susceptibility, 84 were assessed using the E-test method, while only one isolate was tested using the disc diffusion method. Overall, 48.2% were susceptible to the agent, with a median MIC of 0.064 (0.047–0.094) µg/mL. A total of 45.9% of the strains exhibited intermediate susceptibility, with a median MIC of 0.250 (0.190–0.750) µg/mL, while 5.9% were resistant to penicillin (median MIC of 3.000 µg/mL). Moreover, 83 isolates were evaluated using the ceftriaxone E-test, while two isolates were examined using the disc diffusion method. A total of 96.5% of the bacteria remained susceptible to ceftriaxone, with a median MIC of 0.380 (0.110–0.500) µg/mL, as illustrated in Tables 4 and S2. S2 Table shows a significant decrease in penicillin susceptibility over the past 4 years (2020–2023), whereas the AST profiles for other agents remained consistent over time. Notably, the average penicillin MICs of the *S. suis* strains identified between 2020 and 2023 were significantly greater than those reported in the previous 12 years (as illustrated in Fig 1A). Furthermore, when the Spearman correlation method was used to analyze the trend of penicillin MICs over time, it was discovered that there was a significant increase in the trend of penicillin MICs during the study period (*p* < 0.001), as demonstrated in Fig 1B.

**Table 4:** Antimicrobial susceptibility profiles of *S. suis* isolates throughout the study period.

Antimicrobial agent (n)	Susceptible, n (%)	Intermediate, n (%)	Resistant, n (%)
Penicillin (85)	41 (48.2)	39 (45.9)	5 (5.9)
median penicillin MICs, µg/mL (IQR) (84)^a^	0.064 (0.047-0.094)	0.250 (0.190-0.750)	3.000 (3.000-6.000)
Ceftriaxone (85)	82 (96.5)	3 (3.5)	0 (0.0)
median ceftriaxone MICs, µg/mL (IQR) (83)^b^	0.380 (0.110-0.500)	1.500 (1.500-2.000)	
Clindamycin (84)	1 (1.2)	0 (0.0)	83 (98.8)
Erythromycin (84)	0 (0.0)	2 (2.4)	82 (97.6)
Ofloxacin (84)	81 (96.4)	1 (1.2)	2 (2.4)
Tetracycline (84)	4 (4.8)	1 (1.2)	79 (94.0)
Vancomycin (85)	85 (100)	0 (0.0)	0 (0.0)

**Abbreviations**: DD, disc diffusion; IQR, interquartile range; MICs, minimum inhibitory concentrations

^a^Penicillin MICs were tested using the E-test method. CLSI defines penicillin MIC breakpoints as: ≤ 0.12 µg/mL (susceptible); 0.25-2 µg/mL (intermediate); ≥ 4 µg/mL (resistant), excluding one isolate tested by the DD method.

^b^Ceftriaxone MICs were tested using the E-test method. CLSI defines ceftriaxone MIC breakpoints as: ≤ 1 µg/mL (susceptible); 2 µg/mL (intermediate); ≥ 4 µg/mL (resistant), excluding two isolates tested by the DD method.

We identified three *S. suis* strains with ceftriaxone-intermediate susceptibilities from three patients. One patient was diagnosed with mitral valve endocarditis, spondylodiscitis, and spinal epidural abscess due to the strain exhibiting a ceftriaxone MIC of 2.0 and a penicillin MIC of 0.25 µg/mL. Combining penicillin with gentamicin for 4 weeks resulted in a favorable outcome. Another patient, also diagnosed with mitral valve endocarditis, had a strain exhibiting a ceftriaxone MIC of 1.5 and a penicillin MIC of 3.0 µg/mL. Vancomycin was administered for 4 weeks, and mitral valve replacement surgery resulted in a favorable outcome. Another case ended fatally from mitral valve endocarditis complicated by a large cerebral infarction with hemorrhagic transformation from the strain exhibiting a ceftriaxone MIC of 1.5 and a penicillin MIC of 0.25 µg/mL.

## Discussion

This study demonstrated that *S. suis* often causes bacteremia, meningitis, and endocarditis in humans, especially in adult males. Only one-third of patients had apparent exposure to pig or pig-derived products before onset. Among infectious complications, multiorgan dysfunction was the leading cause of fatal outcomes, accounting for 32.5% of cases. During the 16-year study, *S. suis* human isolates presented increased penicillin MICs and resistance to various nonbeta-lactam antimicrobials. This trend highlights the need to consider a suitable antimicrobial option for treating this severe infection.

Most of our cases of *S. suis* infection were observed in late adults, with an average age of 59.29 years. Only 19.8% of the cases were associated with alcoholism, and 39.5% of the cases had no underlying medical conditions. However, contrary to these findings, several studies have shown that *S. suis* infection commonly affects healthy young to middle-aged adults, particularly those with a history of alcohol consumption [5,6,8,9,16]. Approximately one-third of patients were at potential risk or had been exposed to pig or pig-derived products. This finding is comparable to the 30–40% of cases in which people are exposed to the consumption, handling, slaughter, and farming of pigs [5,17,18]. The rate and type of exposure vary based on country and socioeconomic status. For example, occupational exposure is greater in industrialized countries, whereas consuming contaminated pork or products is more common in Asian countries [5]. Nevertheless, the exposure rate is often underreported because it is frequently unknown or not recognized by patients (65.0%).

*S. suis* infection typically presents with fever [10,19]. The onset of this infection occurs only a few days after exposure, which is consistent with other research findings [6,20]. *S. suis* can cause various infections, including septicemia, acute meningitis, endocarditis, bone and joint infections, and endophthalmitis. Our study identified two patients who had uncommon infections: pneumonia and spontaneous bacterial peritonitis. A previous study reported that the occurrence rates of pneumonia and peritonitis were 0.3% and 0.4%, respectively [21]. This study identified septicemia as the most common clinical infection, but other studies reported acute meningitis as the leading clinical infection [5,6,12,19]. Among the 34 patients who underwent CSF examination, 26 were diagnosed with acute meningitis based on the study criteria. Only septic patients with clinical presentations suggesting meningitis are suitable candidates for CSF evaluation, while those without a clinical presentation of meningitis may not necessarily need a CSF diagnosis. Thus, in this study, *S. suis*-infected patients whose CSF was examined and confirmed for diagnosis may not represent a true prevalence of meningitis. Other research has shown that endocarditis is one of the factors contributing to the mortality rate of this infection [6]. Studies of *S. suis* endocarditis in Thailand show that *S. suis* infection frequently affects the aortic valve (52%) and the mitral valve (44%). Most of these patients (79%) had no preexisting structural heart disease prior to the onset [22,23]. It often causes significant valvular damage, leading to multiorgan complications [22,23]. The present study revealed that endocarditis is more common among people infected with *S. suis* and MOD. However, the multivariate analysis revealed no significant correlation between MOD and endocarditis, most likely because of our limited sample size. Additionally, the true prevalence of *S. suis* endocarditis may be underestimated since our institute could not conduct echocardiographic studies in all cases of *S. suis* bacteremia. These issues also represent the limitations of this study.

The complications caused by *S. suis* infection can be severe and may include MOD, cardiopulmonary failure, renal failure, septic shock, and disseminated intravascular coagulation. These findings are consistent with those of other studies [5,8,11,21]. Symptoms of respiratory distress and high SOFA scores are strongly associated with MOD and often lead to fatal outcomes. Therefore, patients exhibiting these significant parameters should prompt early antimicrobial treatment and close monitoring to prevent organ dysfunction. Notably, MOD occurred at a lower rate in patients with *S. suis* meningitis than in patients with other clinical manifestations (*p* = 0.006). Previous studies have indicated that patients with meningitis tend to have lower death rates than those with other invasive *S. suis* infections [5,8,10]. Moreover, the fatality rate attributable to *S. suis* meningitis is lower than that attributable to meningitis caused by other bacteria [10]. Meanwhile, those with endocarditis showed a significant association with MOD (Table 3). We believe *S. suis* endocarditis has high rates of both valvular and paravalvular damage, including systemic complications [22,23], which could potentially cause MOD. Nevertheless, the mechanism responsible for the difference between the affected sites of *S. suis* infection and other etiologic agents requires further investigation. Hearing impairment is a common neurological consequence reported in various studies. The prevalence of hearing impairment may be as high as 50–70% among cohorts with *S. suis* meningitis [10,20,21,24]. However, in the overall cohort, the incidence rate of hearing impairment was lower, ranging from 10–30% among all patients [8,25]. A recent clinical trial revealed that dexamethasone to treat meningitis caused by S. *suis* was significantly associated with reduced deafness [26]. The efficacy of dexamethasone therapy in preventing hearing impairment was inconclusive in this study, as the outcomes of seven individuals who received the therapy were not fully documented. In Thailand, the mortality rate of *S. suis* infections ranges from 9.5% to 19.5% [21]. The incidence rates of septicemia and severe complications are similar to those reported in other studies [5,8]. Still, this study’s mortality rate of *S*. *suis*-infected patients was relatively low (7%). This discrepancy could be due to the capacity of patient management in a tertiary care setting and the predominant use of effective antimicrobials, especially ceftriaxone-based regimens.

The primary antimicrobial treatment prescribed in this study was ceftriaxone-based therapy. This treatment included ceftriaxone alone (68.6%) and ceftriaxone in combination with other drugs (8.1%). The typical duration of antimicrobial therapy was 21 days (14–28), consistent with the duration reported in other studies [6,9,12,18]. The duration of treatment depends on the type and severity of the infection, such as septicemia, meningitis, endocarditis, septic arthritis, and spondylitis [6]. Penicillin G has been previously used as a highly effective antimicrobial agent for treating *S. suis* infection. Previous studies have demonstrated that *S*. *suis* isolates are highly susceptible to penicillin (92–100%) [5,8,10,11,13,15,25]*,* with average MICs ranging from 0.015 to 0.06 µg/mL [6]. The current study shows that *S. suis* is becoming less susceptible to penicillin. Specifically, only 48.2% of the bacteria tested were susceptible to penicillin, with a median MIC of 0.064 µg/mL. The results also revealed that over the last 4 years (2020–2023), there has been a trend toward reduced penicillin susceptibility in *S. suis*, with increasing MICs. Another study confirmed these findings, showing that contemporary isolates of *S. suis* have significantly higher penicillin MICs than older isolates [27]. One possible reason for this change could be a substantial increase in penicillin nonsusceptibility among *S. suis* strains isolated from pigs [15,28]. In Thailand, pigs are a common reservoir for *S. suis*, which has developed resistance to multiple antimicrobial agents, such as penicillin, macrolides, tetracycline, and clindamycin [15]. The high prevalence of antimicrobial resistance in *S. suis* strains carried by pigs poses a significant risk of transmitting resistant bacteria to humans, potentially leading to infections. Recently, an outbreak of *S*. *suis* occurred in northeastern Thailand, resulting in the unfortunate deaths of two individuals [29]. A study of this outbreak revealed that the strains involved are closely related to multidrug-resistant zoonotic strains that have emerged in Thailand over recent decades [29]. In Thailand, most antibiotics do not require a prescription and can be dispensed by licensed pharmacists or veterinarians. Consequently, farmers have easy access to the overuse of antibiotics. A cross-sectional survey revealed that three-quarters of pig farmers reported using certain antibiotic-active ingredients for disease prevention, such as amoxicillin (39.6%), enrofloxacin (22.9%), tetracycline (12.5%), combinations of penicillins with other antibacterials (12.5%), and lincomycin (10.4%). About 46% of the unknown active ingredients were used [30]. The extensive use of antimicrobials in farming and livestock may increase the pressure on *S*. *suis* populations, leading to evolving antimicrobial resistance among these organisms [31]. More than 95–100% of *S. suis* human isolates are still susceptible to extended-spectrum cephalosporins and fluoroquinolones, as reported by several studies [5,8,13,27,29]. According to recent studies and reports, it is crucial to consider the appropriate antimicrobial agent when treating human infections caused by *S. suis*. With an increasing trend of penicillin nonsusceptibility, the use of cephalosporins such as ceftriaxone is recommended, especially in severe cases, while waiting for the results of antimicrobial susceptibility testing. In addition, non-beta-lactam antibiotics, such as vancomycin and fluoroquinolones, which show good *in vitro* activity against penicillin non-susceptible strains based on our study and other reports [13,15], should be considered alternative agents for treating invasive infections in cases of a previous history of severe beta-lactam allergy. Interestingly, we identified three *S. suis* strains with reduced susceptibility to ceftriaxone, indicating that this agent may not be an effective antimicrobial for treating invasive *S. suis* infections, particularly meningitis. Other options, such as carbapenems or vancomycin, should be considered as alternatives for treating ceftriaxone-nonsusceptible strains. Additionally, the change in ceftriaxone susceptibility among *S. suis* requires further monitoring to ensure the selection of appropriate antimicrobial treatment against evolving AMR strains.

This study has several limitations. This observational study collected and reviewed data for 16 years but included only 86 cases. The prevalence of human cases of S. *suis* infection in the central region is lower than that in Thailand’s northern and northeastern regions [7]. Additionally, a retrospective study may only comprehensively evaluate some critical data. Consequently, crucial information, such as potential risks or exposure, long-term complications and outcomes, and even epidemiologic investigations of sources and infected individuals, might have been omitted. We could not analyze the factors that may predict this infection’s prognosis because of the small sample size and number of deaths. The number of *S. suis* isolates studied for antimicrobial susceptibility is relatively low and may not represent the actual AST profile for the year of study. Moreover, most AST data were collected from isolates in the central region, which may only apply to some areas because of differences in local antibiograms. Unfortunately, as a retrospective chart review, we could not thoroughly investigate the genetic determinants of drug resistance and the correlation between antimicrobial-resistant human and pig strains, which may be likely the sources of resistant strains. Therefore, it is necessary to conduct large-scale or nationwide prospective research to study molecular epidemiology and monitor the evolving trends in the antimicrobial susceptibility of *S. suis* in Thailand.

## Conclusion

*S. suis* causes a variety of severe infections and complications, which can lead to life-threatening outcomes. Our study revealed that *S. suis* has increased nonsusceptibility to penicillin and resistance to several other antimicrobial agents. According to the current study, it is crucial to consider the appropriate empirical antimicrobial agent when treating severe *S. suis* infections. Cephalosporins, such as ceftriaxone, or non-beta-lactams, such as vancomycin in cases of prior severe beta-lactam allergy, are recommended, especially for severe cases, while awaiting the results of antimicrobial susceptibility testing. This result highlights the importance of using effective antimicrobial therapy and closely monitoring the emergence of antimicrobial resistance in human infections.

## Materials and methods

### Ethics statement

The Siriraj Institutional Review Board of the Faculty of Medicine Siriraj Hospital, Mahidol University, Bangkok, Thailand approved the study protocol (approval number 051/2022). As the study involved analyzing existing data, the committee waived the requirement for subject consent.

We retrospectively reviewed the medical records of adult patients diagnosed with *S. suis* infection in any part of their body at Siriraj Hospital, Bangkok, Thailand. This review covers the period from May 2007 to May 2023. The data we collected included patients’ demographic information, comorbidities, potential risk and exposure, timing of infection onset and hospitalization, clinical manifestations, severity, laboratory findings, antimicrobial administration, management, and outcomes. Our investigators thoroughly reviewed all medical records, including antimicrobial susceptibility testing (AST) results of *S. suis* isolates during the study period.

### Microbiological methods

When clinical samples such as blood, cerebrospinal fluid (CSF), tissue, or synovial fluid were collected, they were plated on standard culture media. If there were gram-positive cocci in pairs and chains with alpha-hemolysis grown on sheep blood agar plates, the bacteria were identified by biochemical testing and the semiautomated Vitek system (BioMérieux SA, Marcy l’Etoile, France). When samples other than blood cultures, such as tissue biopsies and body fluids, showed no bacterial growth on standard cultures, direct bacterial identification from those residual samples was performed as requested, using polymerase chain reaction and the 16S rDNA sequencing method. Serotyping of *S. suis* isolates was not performed because the necessary test was unavailable at our institute.

E tests (BioMérieux SA, Marcy l’Etoile, France) were used to determine the minimum inhibitory concentrations (MICs) of penicillin and ceftriaxone for the AST of *S. suis* isolates, following the manufacturer’s instructions. On the other hand, clindamycin, erythromycin, ofloxacin, tetracycline, and vancomycin were tested via the disc diffusion method (Oxoid Thermo Fisher Scientific, Basingstoke, UK), following the Clinical and Laboratory Standards Institute (CLSI) guidelines [32]. However, as there were no established interpretative breakpoints for the AST of *S. suis*, the CLSI breakpoint criteria for viridans group streptococci were used for this organism. The CLSI breakpoint criteria for the agents tested were as follows: penicillin MICs of ≤0.12 were considered susceptible, those between 0.25 and 2 were deemed intermediate, and those ≥4 µg/mL were considered resistant. Similarly, those with MICs of ceftriaxone ≤1 were susceptible, those with MICs between >1 and 2 were intermediate, and those with MICs ≥ 4 µg/mL were classified as resistant. In the disc diffusion breakpoint criteria of clindamycin, erythromycin, ofloxacin, tetracycline, and vancomycin, the inhibitory zone size diameters recommended by the CLSI (as defined elsewhere) were utilized [33].

### Definitions

*S. suis* infection was confirmed by positive cultures from various body sites, including blood, CSF, synovial fluid, vitreous fluid, pus, or tissue biopsy. Acute meningitis was diagnosed when a patient showed clinical symptoms and signs of meningeal inflammation; CSF findings suggested bacterial meningitis with positive CSF cultures or additional evidence of *S. suis* infection from other body sites. Septicemia was defined as a positive blood culture for *S. suis*. Endocarditis was diagnosed in patients who met the modified Duke criteria and had a positive blood or tissue valve culture for *S. suis*. The modified Duke criteria for diagnosing infective endocarditis include a positive blood culture, echocardiographic evidence of valvular endocarditis or paravalvular involvement- such as an oscillating mass, vegetation, abscess, or new valvular regurgitation- and findings that meet the minor criteria, as defined elsewhere [34]. Septic arthritis or spondylodiscitis was defined as samples from infected bone or joint being positive for *S. suis*. However, cultures from the affected bones and joints may be negative due to prior antimicrobial treatment and delays in microbiological investigations. Nonetheless, these culture-negative conditions were still considered *S. suis* septic arthritis or spondylodiscitis if *S*. *suis* was identified in blood cultures or other sites. Endophthalmitis was an eye infection with a positive *S. suis* vitreous fluid culture. Skin and soft tissue infections were diagnosed on the basis of clinical signs of local or systemic inflammation and positive cultures of pus or tissue positive for *S. suis*. Symptoms of respiratory distress were defined as the development of signs such as shortness of breath or increased respiratory effort at the onset, including a respiratory rate >22 breaths per minute, rapid and deep breathing, the presence of chest wall retraction, or oxygen desaturation that necessitates oxygen therapy. Patients with multiorgan dysfunction (MOD) were those who exhibited at least two dysfunctions in major organs, such as the kidneys, heart and lungs, and blood clotting. These dysfunctions included acute renal failure, cardiopulmonary failure, disseminated intravascular coagulation, and septic shock. Complete recovery was defined as the resolution of clinical infection without sequelae. Partial recovery with sequelae included partially resolved infections with detected sequelae, such as neurological deficits. The term “death” refers to any cause of death that occurred during hospitalization.

### Statistical analysis

Continuous variables are presented as means and standard deviations (SDs), medians and interquartile ranges (IQR). Categorical variables are expressed as numbers and percentages. Categorical variables were compared using the chi-square test and Fisher’s exact test, whereas continuous variables were compared via an independent t-test or the Mann–Whitney U test. Nonparametric correlation analysis and the Spearman rank correlation coefficient were used to evaluate the relationships between the MICs of the tested antimicrobials and the corresponding year. Univariate and multivariate predictors of multiorgan dysfunction in *S. suis* infection were evaluated using binary logistic regression analysis (forward stepwise method) and presented as odds ratio (OR) (95% confidence interval [CI]). All the statistical analyses were conducted via IBM SPSS 29 Statistics, version 20 (IBM Corp, Armonk, NY, USA). Statistical significance was defined as a *p*-value less than 0.05.

## Supporting information

S1 Table Clinical characteristics and microbiological findings of six *S. suis*-infected patients with fatal outcomes. Abbreviations: CAD, coronary artery disease; DM, diabetes mellitus; HT, hypertension; MIC, minimum inhibitory concentrations; MOD, multiorgan dysfunction; SOFA, sequential organ failure assessment; VAP, ventilator-associated pneumonia. ^a^Cardiovascular instability at the onset is defined as the identification of at least one of the following: systolic blood pressure < 100 mmHg, mean arterial pressure <70 mmHg, or the administration of vasopressors (norepinephrine, epinephrine, dopamine, or dobutamine) required. ^b^Multiorgan dysfunction (MOD) is defined as the identification of at least two organ dysfunctions, such as septic shock, acute renal failure, cardiopulmonary failure, and disseminated intravascular coagulation.
(DOCX)

S2 Table Antimicrobial susceptibility rates for *S. suis* isolates categorized by the year of study. Abbreviations: CLI, clindamycin; CRO, ceftriaxone; ERY, erythromycin; OFL, ofloxacin; PEN, penicillin; S, susceptible; TET, tetracycline; VAN, vancomycin. ^a^84 isolates were tested using the E-test method, while one isolate was tested using the disc diffusion method. ^b^83 isolates were tested using the E-test method, while two isolates were tested using the disc diffusion method. ^c^All isolates were tested using the disc diffusion method.
(DOCX)
