# Supplementary material for: Clinical characteristics and trends in the antimicrobial susceptibility profile of Streptococcus suis infections in a large tertiary hospital, Thailand, 2007–2023
Source: PLoS Negl Trop Dis. 2025 May 19;19(5):e0013110. doi: 10.1371/journal.pntd.0013110 (PMC12124857; doi:10.1371/journal.pntd.0013110)
Supplement: S1 Table — Abbreviations: CAD, coronary artery disease; DM, diabetes mellitus; HT, hypertension; MIC, minimum inhibitory concentrations; MOD, multiorgan dysfunction; SOFA, sequential organ failure assessment; VAP, ventilator-associated pneumonia. aCardiovascular instability at the onset is defined as the identification of at least one of the following: systolic blood pressure < 100 mmHg, mean arterial pressure <70 mmHg, or the administration of vasopressors (norepinephrine, epinephrine, dopamine, or dobutamine) required. bMultiorgan dysfunction (MOD) is defined as the identification of at least two organ dysfunctions, such as septic shock, acute renal failure, cardiopulmonary failure, and disseminated intravascular coagulation. (DOCX) [file pntd.0013110.s001.docx]

**S1 Table. Clinical characteristics and microbiological findings of six *S. suis*-infected patients with fatal outcomes.**

|  | **Patient No. 1** | **Patient No. 2** | **Patient No. 3** | **Patient No. 4** | **Patient No. 5** | **Patient No. 6** |
| --- | --- | --- | --- | --- | --- | --- |
| Gender | Female | Male | Male | Male | Male | Male |
| Age (years) | 61 | 51 | 33 | 46 | 72 | 71 |
| Underlying disease | None | None | Thalassemia,  cirrhosis,  post-splenectomy | Chronic use  of steroid | HT, CAD | HT, DM |
| Onset | 1 days | 7 hours | 1 day | 1 day | 1 day | 3 days |
| Potential risk or exposure | Unknown | Unknown | Unknown | Unknown | Eating raw pork | Unknown |
| SOFA score at the onset | 12 | 11 | 12 | 2 | 5 | 4 |
| Cardiovascular instability  at the onset^a^ | Yes | Yes | Yes | No | Yes | Yes |
| Clinical infection | Septicemia, pneumonia | Septicemia | Septicemia | Septicemia, endocarditis  of mitral valve | Septicemia, septic arthritis | Septicemia |
| Type of primary  antimicrobial therapy | Meropenem | Meropenem | Ceftriaxone | Penicillin G plus gentamicin | Ceftriaxone | Ceftriaxone |
| Penicillin susceptibility test  of the isolate, MICs (µg/mL) | Susceptible,  MICs 0.12 | Susceptible,  MICs 0.064 | Susceptible,  MICs 0.047 | Intermediate,  MICs 0.25 | Susceptible,  MICs 0.047 | Intermediate,  MICs 0.5 |
| Ceftriaxone susceptibility test  of the isolate, MICs (µg/mL) | Susceptible,  MICs 0.125 | Susceptible,  MICs 0.25 | Susceptible,  MICs 0.064 | Intermediate,  MICs 1.5 | Susceptible,  MICs 0.094 | Susceptible,  MICs 0.75 |
| Complications | MOD^b^ | MOD^b^ | MOD^b^ | MOD^b^, a large cerebral infraction  with hemorrhagic transformation | MOD^b^, myocardial  infarction | Renal failure,  cerebral infarction,  severe COVID-19 pneumonia, VAP |
| Length of hospital stay | 2 days | 1 day | 1 day | 4 days | 5 days | 42 days |

**Abbreviations:** CAD, coronary artery disease; DM, diabetes mellitus; HT, hypertension; MIC, minimum inhibitory concentrations; MOD, multiorgan dysfunction; SOFA, sequential organ failure assessment; VAP, ventilator-associated pneumonia

^a^ Cardiovascular instability at the onset is defined as the identification of at least one of the following: systolic blood pressure < 100 mmHg, mean arterial pressure <70 mmHg, or the administration of vasopressors (norepinephrine, epinephrine, dopamine, or dobutamine) required.

^b^ Multiorgan dysfunction (MOD) is defined as the identification of at least two organ dysfunctions, such as septic shock, acute renal failure, cardiopulmonary failure, and disseminated intravascular coagulation.
