# Supplementary material for: Clinical characteristics and trends in the antimicrobial susceptibility profile of Streptococcus suis infections in a large tertiary hospital, Thailand, 2007–2023
Source: PLoS Negl Trop Dis. 2025 May 19;19(5):e0013110. doi: 10.1371/journal.pntd.0013110 (PMC12124857; doi:10.1371/journal.pntd.0013110)
Supplement: S2 Table — Abbreviations: CLI, clindamycin; CRO, ceftriaxone; ERY, erythromycin; OFL, ofloxacin; PEN, penicillin; S, susceptible; TET, tetracycline; VAN, vancomycin. a84 isolates were tested using the E-test method, while one isolate was tested using the disc diffusion method. b83 isolates were tested using the E-test method, while two isolates were tested using the disc diffusion method. cAll isolates were tested using the disc diffusion method. (DOCX) [file pntd.0013110.s002.docx]

**S2 Table. Antimicrobial susceptibility rates for *S. suis* isolates categorized by the year of study.**

| Agent | 2007  (n = 3) | 2008  (n = 6) | 2009  (n = 0) | 2010  (n = 3) | 2011  (n = 0) | 2012  (n = 3) | 2013  (n = 2) | 2014  (n = 12) | 2015  (n = 4) | 2016  (n = 4) | 2017  (n = 2) | 2018  (n = 4) | 2019  (n = 6) | 2020  (n = 2) | 2021  (n = 15) | 2022  (n = 15) | 2023  (n = 4) | Total |
| --- | --- | --- | --- | --- | --- | --- | --- | --- | --- | --- | --- | --- | --- | --- | --- | --- | --- | --- |
|  | n (%S) | n (%S) | n (%S) | n (%S) | n (%S) | n (%S) | n (%S) | n (%S) | n (%S) | n (%S) | n (%S) | n (%S) | n (%S) | n (%S) | n (%S) | n (%S) | n (%S) | n (%S) |
| PEN  (85)^a^ | 3 (100.0) | 4  (66.7) | - | 3 (100.0) | - | 2  (66.7) | 1  (50.0) | 4  (33.3) | 2  (50.0) | 4 (100.0) | 2 (100.0) | 4 (100.0) | 5  (83.3) | 1  (50.0) | 6  (40.0) | 0  (0.0) | 0  (0.0) | 41  (48.2) |
| CRO  (85)^b^ | 3 (100.0) | 5  (83.3) | - | 3 (100.0) | - | 3 (100.0) | 2 (100.0) | 11 (91.7) | 4 (100.0) | 4 (100.0) | 2 (100.0) | 4 (100.0) | 6 (100.0) | 1  (50.0) | 15 (100.0) | 15 (100.0) | 4 (100.0) | 82  (96.5) |
| CLI  (84)^c^ | 0  (0.0) | 0  (0.0) | - | 0  (0.0) | - | 1  (33.3) | 0  (0.0) | 0  (0.0) | 0  (0.0) | 0  (0.0) | 0  (0.0) | 0  (0.0) | 0  (0.0) | 0  (0.0) | 0  (0.0) | 0  (0.0) | 0  (0.0) | 1  (1.2) |
| ERY  (84)^c^ | 0  (0.0) | 0  (0.0) | - | 0  (0.0) | - | 0  (0.0) | 0  (0.0) | 0  (0.0) | 0  (0.0) | 0  (0.0) | 0  (0.0) | 0  (0.0) | 0  (0.0) | 0  (0.0) | 0  (0.0) | 0  (0.0) | 0  (0.0) | 0  (0.0) |
| OFL  (84)^c^ | 3 (100.0) | 6 (100.0) | - | 3 (100.0) | - | 2  (66.7) | 2 (100.0) | 12 (100.0) | 4 (100.0) | 4 (100.0) | 2  (100) | 4  (100.0) | 5  (83.3) | 2  (100) | 14 (93.3) | 15 (100.0) | 4 (100.0) | 81 (96.4) |
| TET  (84)^c^ | 0  (0.0) | 0  (0.0) | - | 0  (0.0) | - | 0  (0.0) | 0  (0.0) | 0  (0.0) | 0  (0.0) | 0  (0.0) | 0  (0.0) | 0  (0.0) | 1  (16.7) | 0  (0.0) | 1  (6.7) | 2  (13.3) | 0  (0.0) | 4  (4.8) |
| VAN  (85)^c^ | 3 (100.0) | 6 (100.0) | - | 3 (100.0) | - | 3 (100.0) | 2 (100.0) | 12 (100.0) | 4 (100.0) | 4 (100.0) | 2 (100.0) | 4 (100.0) | 6 (100.0) | 2 (100.0) | 15 (100.0) | 15 (100.0) | 4 (100.0) | 85 (100.0) |

**Abbreviations**: CLI, clindamycin; CRO, ceftriaxone; ERY, erythromycin; OFL, ofloxacin; PEN, penicillin; S, susceptible; TET, tetracycline; VAN, vancomycin

^a^ 84 isolates were tested using the E-test method, while one isolate was tested using the disc diffusion method.

^b^ 83 isolates were tested using the E-test method, while two isolates were tested using the disc diffusion method.

^c^ All isolates were tested using the disc diffusion method.
